# The Frontier of Entomo-Virology: Applications and Tools for Virus and Vector Surveillance

**DOI:** 10.3390/pathogens14070699

**Published:** 2025-07-15

**Authors:** Poliana da Silva Lemos, Mayron Mielly Morais Pacheco, Bruna Laís Sena do Nascimento, Mônica Silva Coelho, Luciano Chaves Franco Filho, Daniel Damous Dias, Leonardo Sena, Sandro Patroca da Silva, Maria Anice Mureb Sallum

**Affiliations:** 1 Department of Epidemiology, School of Public Health, University of Sao Paulo, Sao Paulo 01246-904, Brazil; mayronpacheco01@gmail.com (M.M.M.P.); masallum@usp.br (M.A.M.S.); 2 Arbovirology and Hemorrhagic Fevers Section, Evandro Chagas Institute, Ananindeua 67030-000, Brazil; brunanascimento@iec.gov.br (B.L.S.d.N.); damous1994@gmail.com (D.D.D.); spatroca@gmail.com (S.P.d.S.); 3 Institute of Biological Sciences, Federal University of Para, Belem 66075-110, Brazil; mocoelho@yahoo.com.br (M.S.C.); lsena@ufpa.br (L.S.); 4 Bacteriology Section, Evandro Chagas Institute, Ananindeua 67030-000, Brazil; lucianofranco6@gmail.com

**Keywords:** entomo-virology surveillance, arboviruses, mosquito

## Abstract

The term ‘entomo-virology’ arose because of the confluence of entomology and virology, focused on deepening the knowledge about the interactions between vectors and viruses and the aspects that involve hosts and the environment. Based on this, entomo-virological surveillance was proposed, aiming to develop tools that strengthen prevention for arboviral disease and vector control strategies. This review aims to present a narrative synthesis regarding the component elements of the concept of entomo-virology. In addition, the applications and tools for the surveillance of viruses and vectors, their implementation challenges, and perspectives are discussed.

## 1. Introduction

Vector-borne diseases pose a significant burden on global health. More than 80% of the world’s population lives in regions where at least one vector-borne disease is endemic. Prominent examples of vector-borne diseases include malaria, dengue, lymphatic filariasis, schistosomiasis, chikungunya, onchocerciasis, Chagas disease, leishmaniasis, Zika virus disease, yellow fever, and Japanese encephalitis [1].

Arboviral diseases are a subset of vector-borne diseases caused by viral pathogens transmitted by arthropod vectors to vertebrate hosts. These viruses belong to diverse taxonomic families, including *Togaviridae*, *Flaviviridae*, *Peribunyaviridae*, and *Rhabdoviridae* [2]. Hematophagous arthropods such as mosquitoes, ticks, sand flies, and midges are vectors of several arboviruses, which can be transmitted to susceptible vertebrate hosts and cause diseases. Arboviral diseases are acute febrile illnesses with a wide range of clinical manifestations, from mild fever to severe complications such as neurological disorders, shock, congenital anomalies, and hemorrhagic fever. Fatal outcomes are not uncommon [3]. These diseases pose a significant global health challenge, with dengue alone accounting for an estimated annual economic burden of USD 8.9 billion [4]. The lack of effective vaccines against most arboviruses and specific treatments highlights the critical importance of robust surveillance and vector control strategies as the primary tools for preventing and mitigating arbovirus outbreaks [3,5].

Despite the implementation of control measures in numerous countries, the desired outcomes have often fallen short [1]. This shortfall is largely due to the lack of comprehensive policies that account for the territorial, demographic, and ecological factors shaping host–pathogen–vector interactions [1,6]. Furthermore, gaps in understanding the biology, diversity, and ecological dynamics of vector species undermine predictive modeling efforts and complicate the design and implementation of effective control strategies.

This narrative review provides an overview of entomo-virological surveillance, especially focused on mosquitoes, encompassing prevention and vector control strategies for arboviruses, in the context of advancements in virus and vector surveillance tools. We included articles published between 1 January 1994 and 30 December 2024. The literature search encompassed English, Spanish, and Portuguese publications, including peer-reviewed articles, systematic reviews, official documents, randomized controlled trials, and observational studies that utilized entomo-virological approaches for arbovirus surveillance, diagnosis, and prevention. Scientific articles not published in English, Spanish, and Portuguese, case studies, and articles that do not discuss the relationship between arboviruses and mosquitoes were excluded. This review aims to inform a diverse audience, including entomologists, virologists, public health managers, academics, and health professionals.

## 2. What Is Entomo-Virological Surveillance?

The molecular detection of arboviruses in arthropod vectors, including mosquitoes, biting midges, and ticks, is a critical component of entomo-virological surveillance. This approach involves collecting vector samples from the field and analyzing them to identify the circulation of arboviruses in the environment, enabling the early detection of potential outbreaks. It also plays a critical role in monitoring the geographic distribution and diversity of arboviruses, which is essential for designing targeted control strategies. A notable example is a study carried out in Greece by Tsioka et al. [7], which demonstrated the effectiveness of entomo-virological surveillance detecting West Nile virus (*Orthoflavivirus nilense*—WNV) in mosquitoes two weeks prior to the first reported human case. In regions where arboviral transmission is sporadic or produces mild symptoms, the importance of early detection becomes even more pronounced. Research conducted in India highlighted this utility when Zika virus (*Orthoflavivirus zikaense*—ZIKV) was detected in *Aedes aegypti* mosquitoes, emphasizing the vector’s pivotal role in the 2016–2021 ZIKV epidemic [8], as well as in infections detected in Brazil [9]. Chikungunya virus (*Alphavirus chikungunya*—CHIKV) and dengue virus (*Orthoflavivirus denguei*—DENV) were also detected using the entomo-virological approach, reinforcing the importance of monitoring *Ae. aegypti* as the main arbovirus vector in urban areas of Brazil [10,11,12,13]. Studies demonstrated the importance of the entomo-virological approach by analyzing data from the largest outbreak of sylvatic yellow fever virus (*Orthoflavivirus flavi*—YFV) in Brazil between 2016 and 2018, demonstrating that not only the primary vectors *Haemagogus janthinomys* and *Hg. leucocelaenus* were involved in the transmission cycle, but several other species recorded YFV detection and thus must be closely monitored, such as *Sabethes chloropterus*, *Ae. albopictus*, *Ae. scapularis*, and *Ae. serratus* [14,15,16,17,18,19].

Entomo-virological data can incorporate mathematical models to predict arboviral outbreak risks, identify key drivers of viral transmission, and evaluate the environmental factors influencing viral dissemination. This integrative approach has been validated through several studies, further illustrating its potential to enhance public health preparedness and response [20,21,22,23]. Entomo-virological surveillance is also a fundamental component of integrated vector management (IVM), a comprehensive strategy that employs a combination of interventions to optimize urban vector control. The selection of specific interventions within IVM is guided by factors such as the nature of the vector-borne disease problem, cost-effectiveness, ecological sustainability, and available resources [24]. The urgent need for implementing IVM is underscored by the emergence of insecticide resistance, as well as the globalization-driven introduction of novel vectors and pathogens. Expanding the application of molecular protocols in entomo-virological surveillance could, in due course, bolster the monitoring of insecticide resistance. Once laboratory infrastructure and workflows are established, these protocols could facilitate the mapping of resistance mechanisms within vector populations. This includes, for instance, the identification of *kdr* mutations, the detection of P450 gene overexpression, and/or the analysis of chitin synthase I1043F mutation, which is associated to diflubenzuron resistance [12,25,26].

## 3. How Do Mosquitoes Participate in the Maintenance and Transmission of Arboviruses in the Environment?

Arbovirus transmission involves a complex mechanism comprising several factors, including a virulent viral strain capable of replicating in both vertebrate and invertebrate hosts, susceptible vertebrate hosts, and competent arthropod vectors. For an arthropod to serve as a vector, it must acquire the pathogen through a blood meal, allow for its replication within its body, and subsequently transmit it to a susceptible host [27]. It is worth noting that pathogen acquisition in vectors can also occur through alternative routes, such as horizontal and sexual transmission, which will be discussed later in this article. A critical aspect of this process is the extrinsic incubation period (EIP), which is the time interval between the arthropod acquiring the pathogen and its ability to transmit it via its saliva. The EIP is influenced by environmental factors such as temperature and humidity, as well as vector-intrinsic factors, including susceptibility to infection and interactions with its microbiota. During a viral infection in mosquitoes, the virus must overcome several barriers. These include successful infection and replication in midgut epithelial cells after a blood meal (midgut infection barrier—MIB), subsequent traversal of the midgut basal lamina to access the mosquito hemocoel (midgut escape barrier—MEB), survival and transportation within the hemocoel to the target tissues, primarily the salivary glands, infection of the salivary gland epithelial cells (salivary gland infection barrier—SGIB), and eventual release into the salivary ducts for dissemination during feeding (salivary gland escape barrier—SGEB) [28,29].

The transmission dynamic of a mosquito arbovirus is primarily shaped by ecological factors, as the cohabitation of mosquitoes and hosts in shared environments enhances transmission opportunities through an increased host–mosquito contact rate [30]. Also, vector longevity is critical, since viruses must complete their replication cycle within the mosquito before being transmitted to a new host [31]. Vector competence, which consists in the intrinsic capacity of a vector to transmit a pathogen considering complex processes and barriers specific to the vector [32], varies significantly among mosquito species and even between populations of the same species [33,34,35,36]. These variations underscore the need to characterize local mosquito populations for accurate arbovirus transmission risk assessments. Furthermore, the appetitive behavior of mosquito populations has been shown to be an important factor in defining their vectorial competence. Recent studies have shown that multiple sequential blood feedings can increase the dissemination of arboviruses in several mosquito genera. This phenomenon may be attributed to several factors, including damage to the midgut basal lamina caused by blood feeding, a reduction in the EIP [37,38,39], or acceleration of parasite development [40,41].

The biological interaction between viruses and their vectors arises from a continuous coevolutionary process. This process is shaped by the vector’s intrinsic factors and environmental interactions, including the microbiota and the presence of insect-specific viruses (ISVs). ISVs are a diverse group of viruses that replicate in insects (in vivo and in vitro) but are incapable of replicating in vertebrates and their cells, being transmitted vertically to progeny in insects [42,43]. These elements, as well as the microbiota, can exert a direct influence on the vectorial capacity of a vector population, defined as the ability of a vector population to transmit pathogens to a susceptible host, providing a more robust framework for understanding its role in pathogen transmission dynamics [44]. One example of the modulation promoted by ISVs is a phenomenon called ‘superinfection exclusion’, whereby a primary ISV infection in an insect inhibits infections by genetically similar or closely related viruses [45]. Another example of interaction occurs in *Ae. aegypti* infected with symbiotic bacteria of the genus *Wolbachia*. The presence of these bacteria inhibits the replication of arboviruses, such as DENV, in *Aedes* sp. mosquitoes, in addition to impacting population density, reducing the longevity of females, and reducing the number of offspring due to cytoplasmic incompatibility between reproductive cells [26,32,46].

Mosquitoes exhibit diverse adaptive strategies to cope with environmental changes, including insecticide resistance, ecological plasticity, dietary shifts, and physiological adjustments. Insecticide resistance has become a growing concern in recent years, with studies highlighting the rapid evolution of resistance mechanisms in mosquito populations, such as target-site mutations and increased detoxification-enzyme activity, which compromise the efficacy of chemical control measures [47,48]. Ecological plasticity enables mosquitoes to exploit a wide range of habitats, from natural to urban environments, allowing species to thrive in densely populated areas with abundant artificial breeding sites [49,50]. Dietary shifts, including increased reliance on human blood meals, have been observed in some mosquito populations, further enhancing their ability to transmit pathogens [51]. Physiological adaptations, such as thermal tolerance, enable mosquitoes to survive in a broader range of climatic conditions, extending their geographic distribution and seasonal activity [52,53].

During adverse conditions such as drought or low temperatures, certain mosquito species, such as those of the tribe Aedini, may enter reproductive diapause, a state of suspended development that enhances survival. For example, observations made with *Hg. leucocelaenus* indicated that eggs were 1.5 times more likely to hatch during the rainy season than during the dry season. This phenomenon may represent an evolutionary strategy that increases the survival rates, since the rainy season provides mosquitoes with greater access to essential resources, including breeding sites, high relative humidity, and greater plant cover and host abundance [54]. Sim and Denlinger [55] identified the molecular and hormonal pathways involved in diapause, offering insights into how mosquitoes synchronize their reproductive cycles with environmental cues. Mosquito eggs also demonstrate remarkable resilience; for example, *Ae. aegypti* eggs can remain viable for over a year in dry conditions, allowing populations to persist and rebound quickly when conditions improve [56,57]. Emerging studies have also reported that these mosquitoes can withstand varying levels of salinity, suggesting an even greater adaptive capacity in response to environmental stressors [58,59,60]. These findings underscore the remarkable adaptability of mosquitoes and the challenges this poses for controlling mosquito-borne diseases in a rapidly changing world.

## 4. How Does the Prolonged Viability of Mosquito Eggs Influence Viral Transmission Dynamics?

Arthropods are the true reservoir of arboviruses. Once infected, arthropods remain infected and may be capable of transmitting the virus to susceptible vertebrates throughout their lives [61]. Arthropods are unable to effectively clear a virus from their bodies, and in some instances, can vertically transmit the virus to their offspring, which results in infected progeny [62].

**Figure 1:**
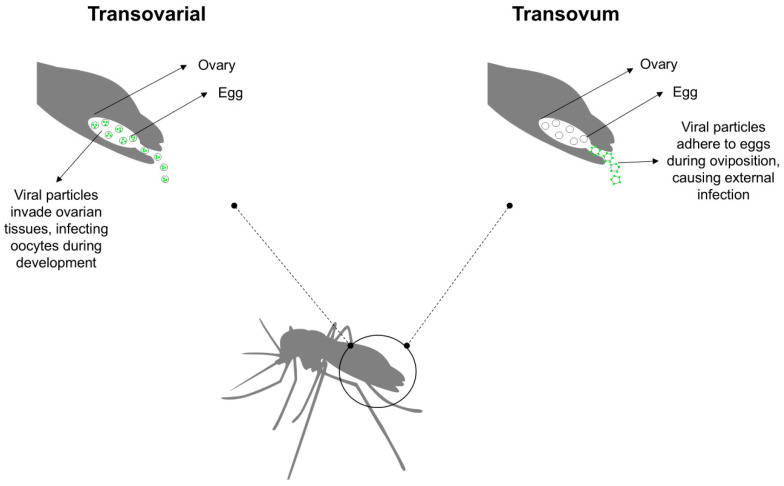
Vertical transmission of viruses in mosquitoes. Viral transmission can occur via two distinct routes: transovarial transmission, where the virus infects the female germline, and trans-egg transmission, a mechanism wherein the virus infects the developing egg during oviposition.

Vertical transmission can occur via two primary mechanisms: transovarial transmission, where the virus infects the female germline, and trans-egg transmission, where the virus infects the developing egg during oviposition (Figure 1) [5,62]. The hypothesis of vertical transmission has been explored in the context of pathogen persistence under adverse environmental conditions, such as drought, winter, interepidemic periods, and intensive vector control measures [62,63]. In this scenario, viruses (arboviruses and ISVs) can persist within mosquito eggs, immature mosquito stages, and adult females, including those entering diapause, without requiring a vertebrate host [5]. The detection of arboviruses in male mosquitoes supports the role of vertical transmission in maintaining arbovirus persistence in the environment. A study conducted in Mexico revealed that 6.7% of the male mosquitoes collected during a post-epidemic period were positive for arboviruses, with CHIKV being the most prevalent (5.7%), followed by DENV (0.9%) and ZIKV (0.1%) [64]. Alencar et al. [65] identified positivity for ZIKV and YFV in samples of male and female *Ae. albopictus* and *Hg. leucocelaenus*, collected through ovitraps, in Rio de Janeiro, Brazil. Although *Culex* eggs exhibit reduced environmental resilience compared to *Aedes* sp. eggs, vertical transmission of arboviruses has been observed in *Culex* species [66].

There is limited information about how these horizontal infection events could trigger new transmission cycles leading to outbreaks or even how this persistence affects the virulence of viruses. Two studies [67,68] demonstrated the horizontal (venereal) transmission of ZIKV in *Ae. aegypti*. In these research works, male mosquitoes (infected through intrathoracic microinjection) transmitted ZIKV to females during copulation. Similarly, female mosquitoes (orally infected) transmitted the virus to males during copulation. Studies on horizontal transmission need to be encouraged, and this information considered, as outbreak forecasting based on traditional epidemiological models that do not incorporate this mechanism may lead to inaccurate predictions.

## 5. Methodologies Applied to Entomo-Virological Surveillance

Vector surveillance methodologies are, in general, a combination of the techniques employed in field entomology, such as the collection of specimens in the field, identification, and sometimes, their maintenance in the laboratory, and virology techniques that, in part, are based on molecular biology and genomics. Studies focused on the analysis of vector competence in mosquitoes may also use techniques of cell culture and viral isolation [34,36,69].

The choice of mosquito collection strategies depends on the surveillance objective. The methodological design should incorporate the biological and behavioral characteristics of the vector species involved in arbovirus transmission. Both active and passive methods can be used to achieve this, including CDC light traps (LT), CDC LT with CO_2_ attractants, human landing catches, ovitraps, BG-Pro sentinels, and larval collection. Comprehensive surveillance or outbreak investigations can benefit from a combination of complementary collection methods to facilitate an extensive sampling of the local vector population. For example, a study conducted in West Africa using nets suspended from helium balloons at altitudes of 120–290 m allowed for the tracking of 61 mosquito species, with some mosquitoes positive for arbovirus, *Plasmodium* sp., and filarial infections [70]. A list of materials, equipment, and biosafety requirements needed to conduct entomological investigations can be found in the Supplementary Materials section of this article.

It is necessary to note that the primary objective of entomo-virological surveillance is the detection of viruses. Consequently, all aspects of field operations, from specimen collection and identification to nucleic acid extraction and subsequent analyses, must be optimized to ensure successful viral detection. For instance, mosquitoes collected using traps should be processed promptly and stored under conditions that preserve the viral genetic material, such as ultra-refrigeration (−80 °C), in liquid nitrogen dewars (−196 °C), or in appropriate preservation media (ethanol, propylene glycol, and nucleic acid preservation reagent) [71,72].

In entomo-virological studies, specimens in a range of life stages can be collected, including eggs [34,69], larvae, pupae [8,35,73], and adults [7,14,66,74,75,76,77,78,79]. Adults are preferentially collected due to the higher likelihood that they offer of detecting viral infections, considering both vertical and horizontal transmission routes. The combination of vertical and horizontal arbovirus transmission in mosquito populations represents a highly effective strategy that promotes the persistence of these pathogens in the environment. These modes of transmission diversify the pathways through which pathogens can spread and endure within a vector population [62]. Furthermore, morphological identification keys are primarily based on adult mosquitoes, particularly females, as many diagnostic morphological characteristics are not fully developed in early larval instars [80]. Collecting immature life stages (eggs, larvae, and pupae) is generally considered to pose lower risks to researchers compared to adult collection methods like human landing catches, Shannon traps, and manual aspiration. Nevertheless, collecting immature stages demands a comprehensive understanding of vector ecology and appropriate facilities to rear specimens to later developmental stages [81]. However, molecular methods that will be mentioned below in this review can be used for the identification of immature specimens, eliminating the need for laboratory rearing. Another interesting biological specimen processed in some studies is the adult mosquito’s excreta. An adaptation of BG-Sentinel traps, developed by Manzi et al. [82], utilizes FTA cards soaked with a solution made of honey and a hydroxycellulose hydrogel. This medium not only ensures the preservation of the viral nucleic acids expelled by mosquitoes onto the card but also remarkably keeps the mosquitoes alive for days, thereby maximizing the collection efficiency. L’Ambert et al. [83] were able to detect WNV circulation in Camargue, France, using a xenomonitoring method based on the molecular detection of the virus in excreta from trapped mosquitoes. This strategy shed light on how viral surveillance can complement standard surveillance methods.

Accurate taxonomic identification is essential for comprehending the dynamics and factors driving arbovirus transmission. Morphological identification remains the gold standard [80]. However, it necessitates the expertise of skilled taxonomists, particularly when rapid viral detection is the primary goal. Identification on refrigerated tables or on chemical ice packs improves the sample preservation conditions, aiming at viral detection or isolation. Innovative approaches have been developed to supplement or improve the accuracy of mosquito species identification. Machine learning algorithms offer a promising avenue for automated morphological identification, although large datasets are required for training the algorithms [84]. Over the past two decades, mitochondrial and ribosomal genes have become established tools for species-level taxonomic identification in entomological research. Commonly employed markers include cytochrome oxidase 1 (COI), cytochrome oxidase B (CytB), ITS2, D2, and 28S and 12S rRNA [80,85,86,87]. DNA barcoding can be integrated with viral detection protocols [85,88,89,90,91], providing a valuable tool for situations where expert taxonomic expertise is limited. However, DNA barcoding-based identification still faces challenges in resolving recent intraspecific divergences [86,92].

Reverse transcription–quantitative polymerase chain reaction (RT-qPCR) has become a cornerstone technique for the direct detection of viruses in mosquito samples within the field of entomo-virology [7,8,14,66,74,77,78,79,89]. While RT-qPCR is often used as a primary detection tool, it is frequently complemented by whole-genome sequencing of positive samples to characterize viral genomes and infer phylogenetic relationships [7,66,74,76,78,89,93]. A critical aspect of RT-qPCR-based arbovirus detection in mosquitoes is the establishment of a standardized threshold for positivity. The cycle threshold (Ct) value, representing the number of cycles required for the fluorescent signal to cross a defined threshold, is commonly used to determine positivity. However, the optimal Ct cutoff can vary depending on factors such as the target virus, sample quality, and assay sensitivity. While some studies have adopted Ct cutoffs of 35 [36], 37 [14], 38 [64,77,93], or even 40 [79], the choice of the cutoff remains a subject of ongoing discussion. For samples with high or indeterminate Ct values, it is recommended to perform replicate RT-qPCR assays using separate aliquots of the extracted RNA to minimize the risk of compromising RNA integrity due to repeated freeze–thaw cycles.

Viral metagenomics offers a novel approach to explore the diverse viral communities associated with mosquito hosts. Unlike targeted RT-qPCR methods, virome analysis provides an unbiased exploration of the entire viral community, including ISVs, viruses associated with the microbiota, and arboviruses [94]. This approach can facilitate the discovery of novel arboviruses with potential public health significance [76]. Virome analysis typically involves next-generation sequencing (NGS) [95] and often leverages high-throughput sequencing platforms [90,96]. While NGS-based virome analysis offers an unparalleled depth of viral discovery, it is a resource-intensive methodology that generates vast amounts of data requiring sophisticated bioinformatics pipelines. Factors such as sample type, collection methods, and sequencing depth can significantly influence the complexity and interpretation of virome data [96]. Although the routine integration of virome analysis into entomo-virological surveillance may be challenging due to technical and logistical constraints, it remains a promising complementary tool for addressing specific research questions and public health concerns.

The positivity of mosquito samples collected from field sites exhibits variability that may be influenced by factors such as vector species/lineage, viral species, and geographic location. For instance, studies investigating WNV surveillance have reported an average positivity rate of 4.71% among mosquito pools comprising *Culex* sp. and *Aedes* sp. in Israel [74]. A comparable positivity rate of 4.4% for WNV-positive pools was documented in Greece [76].

To better assess the transmission risk, entomo-virological studies often employ additional metrics such as the minimum infection rate (MIR) and maximum likelihood estimate (MLE), beyond simple positivity rates [7,8,20,63,66,73,77,78,79,97,98,99,100]. The MIR is determined by dividing the number of mosquito pools positive for arboviruses by the total number of mosquitoes tested and multiplying the result by 1000. The MLE is calculated using the formula [97]:

MLE = [1 − (n − χ/n) 1/m] × 1000

In this equation:

- n = total number of pools tested;
- x = number of positive pools;
- m = pool size.

The MIR provides a conservative estimate of the infection rate, while the MLE offers a more precise estimate of the true infection rate, since it normalizes for the pool size. Under low-intensity transmission scenarios, the MIR and MLE values may be comparable. However, in high-transmission or epidemic settings, these two estimates can diverge significantly [20,101].

However, entomo-virological analyses, even without the taxonomic identification step, can still have important informative value in the context of vector-borne disease surveillance. Although it does not identify the presence of species known to be important vectors, the analysis of large pools with few resources can still inform the presence of the pathogen of interest, serving as a predictor for emergency alert of an outbreak.

Resource limitations are a common challenge for municipalities, making the development of more accessible diagnostic tools crucial. Isothermal amplification technologies, applied to arbovirus detection, offer a timely solution. These methodologies utilize a specific restriction enzyme that functions without thermal cycling. While isothermal amplification may exhibit lower sensitivity compared to diagnostic methods considered gold standards for arboviruses, such as RT-qPCR, its utility as a screening tool in surveillance is significant. When a sample tests positive via this method, it can then be forwarded for more specific and sensitive analyses, optimizing resource allocation and accelerating the response times in public health initiatives [102].

## 6. The Role of Entomo-Virological Surveillance in Understanding the Multi-Vector Transmission of Arboviruses

Upon identifying an arbovirus outbreak or epidemic, immediate preventive actions must be implemented to protect the exposed population. These measures may include vaccination (if available), establishing outpatient and inpatient care services, intensifying laboratory testing, and executing vector control interventions. However, developing a comprehensive prevention and control strategy becomes particularly challenging in urban and rural contexts when multiple vector species are involved in transmitting and maintaining a specific arbovirus. Zoonotic arboviruses that thrive in wild, rural, or suburban environments often rely on multiple vector species and infect a wide range of vertebrate hosts [19,103]. The emergence of such arboviruses at epidemic levels can be triggered by disruptions to natural ecosystems caused by changes in viral genetics, host or vector population dynamics, or anthropogenic environmental modifications [104]. A notable example is the transmission of Oropouche fever in Brazil.

Oropouche fever is a febrile illness in humans caused by the tri-segmented *Orthobunyavirus oropoucheense* (OROV). Its clinical presentation, which includes myalgia, arthralgia, and headache, often overlaps with that of other arboviral infections like dengue. This overlap, coupled with limited diagnostic testing and the co-circulation of multiple arboviruses, contributes to its underreporting [105]. Before the emergence of CHIKV and ZIKV, OROV was the second most prevalent arbovirus in Brazil after dengue. However, a surge in OROV transmission was detected in Brazil starting in 2023, following the implementation of enhanced laboratory testing for cases negative for dengue, chikungunya, and Zika. This heightened surveillance revealed significant OROV circulation, initially concentrated in the Brazilian Amazon (Acre, Amazonas, Rondônia, and Roraima) and later spreading to other regions of the country [106,107].

While *Culicoides paraensis* (*Diptera*: *Ceratopogonidae*) is the primary vector for OROV transmission, other *Diptera* species, such as *Culex quinquefasciatus*, *Aedes scapularis*, *Aedes serratus*, *Coquillettidia venezuelensis*, *Psorophora cingulata*, and *Haemagogus tropicalis*, may also play a role [77,105,107,108,109,110,111]. Additionally, other *Culicoides* species cannot be ruled out as contributors to OROV transmission [112]. Effective prevention strategies for vector-borne diseases, especially those without available vaccines, often begin with vector control measures using insecticides. However, uncertainty surrounding the specific roles of multiple vectors with diverse ecological characteristics increases the risk of implementing ineffective control strategies.

A thorough understanding of vector diversity, competence, and biology is essential to designing targeted control strategies that account for technical, economic, environmental, and public health considerations [113]. Entomo-virological surveillance serves as a critical tool for comprehending the natural history of arboviral diseases and identifying the key factors contributing to the establishment and maintenance of arbovirus transmission. This knowledge is crucial for developing effective preventive measures tailored to the specific epidemiological complexities of each situation.

## 7. Entomology–Virology for Strengthening Border and Port Surveillance

Virological surveillance for arboviruses necessitates prioritizing strategic locations such as ports, borders, and urban centers. These areas function as critical hubs for the movement of goods, people, and potential vectors, creating an environment conducive to the introduction and dissemination of arboviruses. The challenges associated with border regions are further compounded by disparities in healthcare systems, which can impede the prevention and control of emerging health threats [114]. The absence of robust surveillance tools in these strategic locations can compromise preparedness and response efforts in the face of emerging imported pathogens. For instance, DENV and YFV, and likely ZIKV, were introduced into Brazil via ports and cross-border human movement. Airports also play a significant role in the introduction of arboviruses [115,116,117,118]. Travelers can bring new pathogens into a territory. Although the importation of infected vectors is less frequent, it still poses a non-negligible risk. A surveillance program conducted at a major international airport in Central Europe underscored the potential for this route of introduction, revealing high levels of WNV and Usutu virus (*Orthoflavivirus usutuense*—USUV) transmission [66].

## 8. Challenges in Implementing Entomo-Virological Surveillance for Public Health

Implementing a comprehensive public health surveillance strategy necessitates a multifaceted approach that encompasses management, service organization, and robust data infrastructure. While the collection of relevant indicators is a fundamental component, it alone is insufficient for establishing effective entomo-virological surveillance. A systematic framework for data collection, analysis, and response is essential to ensure timely and appropriate interventions.

The COVID-19 pandemic has accelerated the development of genomic surveillance capabilities, particularly within public health laboratories, in many countries, including Brazil [119,120]. Anticipating that climate change may alter the transmission dynamics of arboviruses and increase their impact on human and animal health [121], it is opportune and strategic to leverage these expanded laboratory capacities for integrated arbovirus surveillance [119]. Entomo-virological surveillance represents a strategic approach to expanding the genomic surveillance of arboviruses with public health significance. This approach offers several advantages, including the ability to anticipate outbreaks, understand the epidemic potential of emerging and circulating viral strains, and assess the impact of antiviral drugs and novel vector control strategies [120].

The implementation of entomo-virological surveillance, however, is often hindered by various challenges, including limited human resources, inadequate infrastructure, and difficulties in data collection and sharing. To address these limitations, a comprehensive surveillance strategy should be developed, fostering collaboration among stakeholders and providing opportunities for training and capacity building. Effective health management is essential for consolidating integrated surveillance systems for arboviruses. This involves coordinating efforts with sectors responsible for solid waste management, urbanization, water supply, and environmental education, as well as engaging with the community. By investing in surveillance infrastructure and promoting data sharing, it is possible to strengthen the integration of environmental, epidemiological, and laboratory surveillance. And with this understanding, it is possible to reinforce the importance of directing government investments towards the training of entomologists and the construction of structures that enable the implementation of entomo-virological surveillance as a tool for preventing and responding to the threats that vector-borne diseases represent for public health.

Community communication, education, and engagement are fundamental pillars that support the structure of communicable disease surveillance, with special emphasis on entomo-virological surveillance. This interface is crucial for the effective execution and success of surveillance activities. A participatory community process significantly increases the receptiveness of the population to public health interventions. This direct collaboration facilitates the identification of vector breeding sites, the application of vector prevention and control measures, the detection of suspected human cases, and epizootics [122]. This strategy helps to direct actions and optimize epidemiological and entomological investigations, thus allowing for a faster and more efficient response to public health threats.

## 9. The Arbovirus Diagnostic Laboratory Network of the Americas (RELDA)

The Pan American Health Organization (PAHO/WHO), in partnership with its member states, established the Americas Arbovirus Diagnostic Network (RELDA) to enhance the capacity for arbovirus diagnosis and surveillance. Building upon the successful Dengue Laboratory Network of the Americas, which was launched in 2008, RELDA aims to strengthen laboratory infrastructure, harmonize diagnostic protocols, and promote collaboration among laboratories in the region (https://www.paho.org/en/topics/dengue/arbovirus-diagnosis-laboratory-network-americas-relda (accessed on 27 December 2024)).

The emergence of CHIKV and ZIKV viruses in the Americas highlighted the need for a more robust and coordinated regional response to arbovirus outbreaks. As a result, RELDA expanded its scope to include the Arbovirus Genomic Surveillance Platform (ViGenDa) and the Entomo-Virological Laboratory Network (RELEVA). Currently, RELDA comprises 40 laboratories from across the Americas, including Argentina, Brazil, Canada, and the United States. These laboratories collaborate to improve diagnostic capabilities, share data, and respond to emerging threats. Additionally, five collaborating centers provide technical support and guidance to the network. These centers include the National Institute of Human Viral Diseases in Argentina, the Evandro Chagas Institute in Brazil, and the Centers for Disease Control and Prevention in the United States.

## 10. Conclusions

Entomo-virological surveillance is a necessary tool for assessing risks and identifying the factors that drive arbovirus outbreaks and epidemics. In public health, this type of surveillance can be implemented at various levels, ranging from localized monitoring in specific areas such as ports, airports, and sentinel municipalities to broader, nationwide efforts, depending on the operational resources available.

The transmission dynamics of arboviruses depends on specific environmental conditions, characteristics of the arboviruses itself and its vectors (especially the capacity of being maintained for several months in eggs), and previous contact of human populations with that pathogen, which will affect their susceptibility to disease and symptoms. All factors will influence the risk assessment in a local population.

Several methods for mosquito and arbovirus identification using high-throughput genetic data may contribute significantly to assessing the transmission risk and comparing outbreaks in different areas. However, the conditions needed to achieve the whole potential of those strategies are challenging due to the complex logistics of their routine use.

## Supplementary Materials

The following supporting information can be downloaded at: https://www.mdpi.com/article/10.3390/pathogens14070699/s1. File SI: The Frontier of Entomo-virology: Applications and Tools for Virus and Vector Surveillance.

## Abbreviations

CDC LT	CDC light traps
CHIKV	*Alphavirus chikungunya*
COI	Cytochrome oxidase 1
Ct	Cycle threshold
CytB	Cytochrome oxidase B
DENV	*Orthoflavivirus denguei*
DNA	Deoxyribonucleic acid
EIP	Extrinsic incubation period
IVM	Integrated vector management
MEB	Midgut escape barrier
MIB	Midgut infection barrier
MIR	Minimum infection rate
MLE	Maximum likelihood estimate
NGS	Next-generation sequencing
OROV	*Orthobunyavirus oropoucheense*
PAHO	Pan American Health Organization
RELDA	Americas Arbovirus Diagnostic Network
RELEVA	Entomo-Virological Laboratory Network
RNA	Ribonucleic acid
rRNA	Ribosomal RNA
RT-qPCR	Reverse transcription–quantitative polymerase chain reaction
SGEB	Salivary gland escape barrier
SGIB	Salivary gland infection barrier
USUV	*Orthoflavivirus usutuense*
ViGenDa	Arbovirus Genomic Surveillance Platform
WHO	World Health Organization
WNV	*Orthoflavivirus nilense*
YFV	*Orthoflavivirus flavi*
ZIKV	*Orthoflavivirus zikaense*
