# Supplementary material for: The Frontier of Entomo-Virology: Applications and Tools for Virus and Vector Surveillance
Source: Pathogens. 2025 Jul 15;14(7):699. doi: 10.3390/pathogens14070699 (PMC12299351; doi:10.3390/pathogens14070699)
Supplement: Supplementary file 1 [file pathogens-14-00699-s001.zip › pathogens-3710258-supplementary.pdf]

Supplementary Material

The Frontier of Entomo-virology: Applications and Tools for Virus and Vector Surveillance

This supplementary document details a comprehensive list of materials, field and laboratory equipment, and personal protective equipment (PPE) that may be employed throughout entomo-virological investigations, spanning from vector collection to molecular analyses. The precise configuration and application of these resources are inherently flexible, contingent upon both the specific aims of the investigation and the available operational capacity.

Field Organization and Personal Items:

- GPS device
- Field datasheet/logbook
- Pencils and permanent markers
- Thermo-hygrometer
- Backpack/field bag
- Trash bags
- Toilet paper
- Water bottle/canteen
- Sunscreen
- Pocket knife or camping knife
- Lighter/matches
- Marking tape (e.g., flagging tape) for trail identification (while activity is ongoing in the area)
- Flashlight with spare batteries
- Communication device (two-way radio/mobile phone)
- Clipboard
- Machete with sheath

Personal Protective Equipment (PPE):

- Clothing covering arms and legs (e.g., long-sleeved shirts and long pants)
- Closed-toe boots or shoes appropriate for field activities
- Gaiters (or snake guards/chaps)
- Waders or rubber boots for flooded/swampy environments
- Rain gear/raincoat
- Helmet with face shield
- Visor, hat, or wide-brimmed cap
- Safety glasses/goggles
- Disposable nitrile gloves
- Disposable or reusable (autoclavable) laboratory coats,
- N95/PFF2 surgical masks.

Traps and Collection Devices:

- CDC light traps
- BG-Sentinel traps
- Castro mosquito aspirator
- Battery-powered entomological aspirators (or equivalent automatic aspirator)

- Entomological net
- Rope or string for trap deployment
- Batteries or chargers
- Mobile platform for canopy collection.

Sample Storage:

- Falcon tubes
- Funnels for sample transfer
- Cryotubes (2 mL or 3 mL)
- Labels/labeling tape
- Liquid nitrogen dewar/container
- Liquid nitrogen
- Dry ice in an insulated cooler with lid
- Medical tape (e.g., micropore) for labeling
- Clear adhesive tape for tube sealing
- Tube with RNA later (if applicable).

Sorting and Taxonomic Identification:

- Petri dishes
- Entomological forceps
- Entomological dissecting needle/scalpel
- Round brush n°2 (or smaller)
- Tubes for storing identified samples

Laboratory Reagents and Consumables:

- Viral RNA extraction kits
- TaqMan primers and probes specific to the investigated pathogens
- Reverse transcriptase enzymes
- qPCR master mixes
- Sterile filter tips
- Disposable filter tips for micropipettes (various volumes)
- Microtubes
- 96-well PCR plates and sealant.

Laboratory Equipment:

- Real-time thermocycler
- Refrigerated centrifuge
- Gel electrophoresis system
- Class II biological safety cabinet
- Variable-volume micropipettes
- Refrigerator and freezers (-20°C and -80°C).
